# Empowering minoritized Alabamians screened for lung cancer—The Alabama Lung Cancer Awareness Screening and Education (ALCASE) project

**DOI:** 10.1002/cam4.70213

**Published:** 2024-10-14

**Authors:** Soumya J. Niranjan, Meghan Tipre, Claudia M. Hardy, Tara Bowman, Monica L. Baskin

**Affiliations:** ^1^ Department of Health Services Administration, School of Health Professions University of Alabama at Birmingham Birmingham Alabama USA; ^2^ Division of Hematology and Oncology, School of Medicine University of Pittsburgh Pittsburgh Pennsylvania USA; ^3^ Division of Hematology/Oncology O'Neal Comprehensive Cancer Center University of Alabama at Birmingham Birmingham Alabama USA; ^4^ O'Neal Comprehensive Cancer Center University of Alabama at Birmingham Birmingham Alabama USA

**Keywords:** Community Health Advisors, Equitable cancer screening, Lung cancer screening

## Abstract

**Background:**

In Alabama only 4% of those eligible have been screened for lung cancer. The ALCASE project focused on navigating eligible individuals to lung cancer screening.

**Methods:**

Trained local staff enrolled screen eligible individuals from seven rural counties and one urban county. Demographics and knowledge of and barriers to lung cancer screening were collected using questionnaires. Education was provided and individuals were navigated to undergo screening. Descriptive statistics for enrolled and screened participants were computed using SAS 9.4. Debriefing interviews were conducted with the ALCASE staff regarding facilitators/barriers to implementing this project and lessons learned. Using NVivo, themes were identified through a combined deductive and inductive process.

**Results:**

In total, 447 people were contacted of which 257 were enrolled. Participants were predominantly African American (86.8%), female (56.8%), and 86.4% had health insurance. Study participants acknowledged the need for more education of lung cancer/screening procedures; help navigating clinics for screening services and having healthcare facilities close to home. The top five barriers to getting screened were transportation, financial issues, emotional concerns, healthcare insurance, and COVID‐19. Of the 257, 106 participants (41%) completed a primary care referral and were screened for lung cancer. Debriefing interviews revealed: (i) Overall impressions of implementing ALCASE were positive. (ii) Barriers in implementing ALCASE were identified predominantly at the physician and institutional level. (iii) Facilitators in implementing ALCASE were identified at multiple levels. (iv) Suggestions on improving lung cancer screening leaned toward mitigating barriers at the institutional and structural level.

**Conclusion:**

Ability to get screened is severely challenged by both personal and structural barriers.

## 1 INTRODUCTION

Lung cancer continues to be the leading cause of cancer mortality in the United States.^1^ It accounts for over 20% of the cancer deaths annually.^2^ This mortality has two main drivers, late stage at initial diagnosis and poor overall survival.^3^ Although novel therapies have increased life expectancy, disease prognosis still largely depends on tumor stage at diagnosis^4^—patients with localized non‐small cell lung cancer have a 5‐year survival rate of 61%, whereas it is 6% in those with metastatic disease.^5^ Thus, the key to lowering mortality from lung cancer is twofold, minimizing risk by identifying modifiable risk factors and early detection through screening.^2^ Lung cancer screening utilizing low‐dose CT (LDCT) has demonstrated a 20% reduction in lung cancer mortality among adults 55 to 74 with a 30 pack‐year smoking history.^6^ Based on this evidence, the US Preventive Services Task Force (USPSTF) recommended screening for asymptomatic adults 55 to 80 years of age^7^ which have since been updated in 2021 to include individuals aged 50 to 80 with a 20 pack‐year smoking history who are current smokers or have quit within 15 yrs.^8^

However, lung cancer screening continues to be underutilized nationally, with less than 6% of those eligible being screened.^9^ Specifically, in Alabama, the rates of lung cancer screening from a recent registry study show an abysmal rate of 1.7%.^10^ Considering that increasing awareness around early detection of lung cancer is urgent and crucial, we aimed to reach underserved, at risk Alabamians. In this project, titled Alabama Lung Cancer Awareness, Screening, and Education (ALCASE), we utilized our well accepted and long‐term Community Heath Advisor (CHA) model and the Deep South Network^11^, ^12^ to promote education and awareness. The ultimate goal of this project was the utilization of a CHA model to identify and navigate 250 screen eligible individuals across seven counties to lung cancer screening. Here, we present the results of this program and lessons learned in implementing this project.

## 2 METHODS AND MATERIALS

### 2.1 Study procedures and study population

Using a “train the trainer” approach, county coordinators trained local, volunteer CHAs to educate, identify and recruit individuals from rural counties (Choctaw, Dallas, Greene, Hale, Marengo, Pike and Sumter) and one urban county for comparison (Jefferson). Counties were defined as rural if they had a Rural–Urban Continuum Code (RUCC) between 4 and 9 and urban if they had a RUCC code between 1 and 3.^13^ Eligibility criteria were based on the original USPSTF lung cancer screening guidelines and included (i) individuals aged 55 to 80 years and in fairly good health; (ii) currently smoking or have quit within the past 15 years; and (iii) have at least a total of 30‐pack‐year smoking history.

#### 2.1.1 Recruitment, education, and baseline assessment

Once a potentially eligible individual was identified, coordinators administered a baseline assessment, facilitated the educational sessions, and consented individuals for navigational support.

Individuals were administered a detailed questionnaire on demographics and needs about lung cancer screening including education knowledge, access to healthcare and healthcare experience, financial assistance, or social needs (Data S1). Most of the information was collected using multiple choice questions with additional option to provide open‐ended responses as needed.

The education curriculum was delivered by county coordinators included screening guidelines issued by the USPSTF and American Cancer Society in addition to updated materials on healthy lifestyles, nutritional advice, smoking cessation, and weight management. Further details about the curriculum and training of CHAs are provided elsewhere.^14^

#### 2.1.2 Follow‐up and navigation of participants for lung cancer screening

After completion of enrollment, baseline assessment, and education, coordinators followed up with the participants to navigate them for lung cancer screening. All lung cancer screenings require referral from primary care providers. Coordinators engaged with participants to facilitate appointments with their primary care providers when assistance was needed. This also included identifying a primary care provider in cases where the individual did not have one. They also provided additional navigational support by assisting with transportation to primary care provider appointments or assistance with enrollment in Medicaid or Medicare insurance. Once a referral was obtained, coordinators followed up with the participants to obtain information about the screening appointment, and location. All lung cancer screenings were undertaken at the nearest Screening Center of Excellence (SCOE). Once the screening was completed, coordinators contacted the participants to obtain the results of the screening and any assistance needed for facilitating follow‐up in case of nodule findings. Overall, coordinators provided assistance with transportation, enrollment in health insurance programs, access to primary care providers, helping patients with appointment reminders, transportation for lung cancer screening appointments, and co‐pays for the screening. Data on barriers to any aspect of the screening navigation were recorded by the coordinators.

Data were collected for this study between June 2018 and December 2021. It is to be noted that the guidelines were updated in March 2021 to lower the age (50 years) and pack‐year history (20‐pack‐year history).^8^ Each participant was provided with a $40 gas card once they scheduled the screening appointment.

*Data analysis*: All data were entered in RedCap and analyzed using SAS Institute Inc. 2016. SAS® 9.4. Data analyses began with cleaning and identifying errors in data entry, and missing information. Based on the responses, health insurance was collapsed into Medicare, Medicaid, Private, Other, or None categories. All other variables remained unchanged. Coordinators recorded barriers to lung cancer screening at each time point including prior to primary care provider appointment, and screening appointment in a redcap form that are multiple choices as well as text fields to list any other barriers that were not listed. In addition to existing options, additional categories were created for Barriers to Screening including “COVID‐19,” “Eligibility Requirement,” and “Phone Issues.” Descriptive statistics were computed using frequencies and proportions for categorical variables and means and standard deviation for continuous variables. Descriptive statistics for demographic variables and barriers experiences were compared between those who consented but did not undergo screening vs. those who underwent lung cancer screening using chi‐square or Fisher's exact statistic.

*Key informant interviews*: Debriefing interview sessions were conducted by SJN (a Medical Sociologist with extensive qualitative methodology expertise^15^, ^16^, ^17^, ^18^, ^19^, ^20^) with five ALCASE staff (three county coordinators, one case manager, and one program director) at the end of the study period. SJN was not involved in program implementation. The case manager was responsible for recruiting, training all CHAs and problem‐solving issues related to the project. The program director provided overall administrative leadership, development of curriculum and ensured accomplishment of goals. A semi‐structured interview guide was created to obtain overall experiences, impressions, and lessons learned. Each individual session lasted between 60 and 90 minutes and the qualitative data analyzed for the evaluation consisted of verbatim transcripts from the interviews. The transcribed interviews were coded according to themes by a single investigator (SJN) using NVivo, a software program designed for qualitative analysis. These themes were identified through two‐stage process including deductive themes based in part on the semi‐structure interview guide and inductive themes that emerged from the content of the interviews.^21^ All study activities commenced after approval from the UAB Institutional Review Board for Human Use, and written consent was obtained for all participants.

## 3 RESULTS—DEMOGRAPHICS, NEEDS, BARRIERS, AND SCREENING RESULTS

**FIGURE 1:**
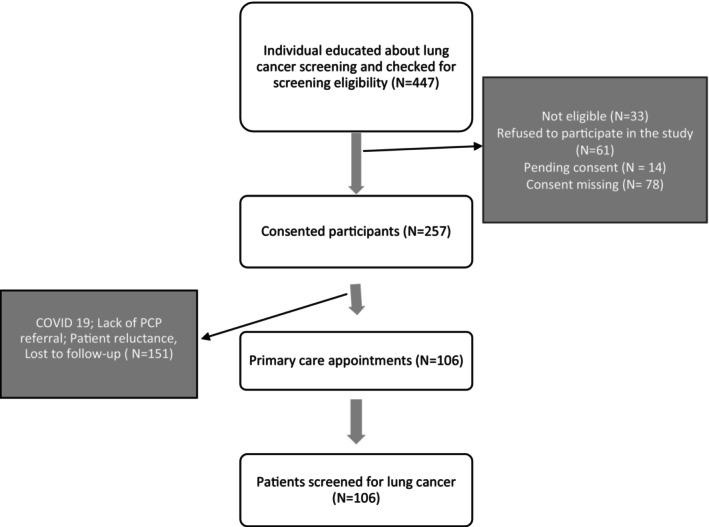
Flow Diagram of Engagement and Recruitment of ALCASE Participants.

Over a period of 2 years (a majority of which was during the early phase of the COVID pandemic), we identified 447 participants. We excluded individuals who were ineligible for lung cancer screening (*N* = 37); or did not consent (*N* = 153). This resulted in 257 individuals who consented to participate in the study. Of these 257 individuals, COVID‐19 restrictions, lack of PCP referral, personal reluctance, and lost to follow‐up resulted in a final cohort of 106 individuals who underwent screening for lung cancer (Figure 1). Participant demographic characteristics of those enrolled in the study and those who underwent screening are presented in Table 1. Enrolled participants ranged between the ages 50 to 84 years with an average age of 62.7 years. Comparing across age groups, about 33.5% of those consented were grouped in age group 50–59 years (*n* = 86), 44.4% in 60–69 years (*n* = 114), 16% in 70–79 years (*n* = 41), and 0.8% (*n* = 2) in 80 to 89 years, respectively. Age was missing for 14 participants (5.4%). Majority of the participants contacted were Black or African American (86.7%) and female (56.8%). Participants reached resided in seven rural counties and one urban comparison county—primarily underserved, predominantly African American areas with typically higher smoking rates and greater than average mortality from lung cancer. About 49.4% of participants resided in the urban county while 48% resided in non‐urban and rural counties.

**TABLE 1:** Characteristics of ALCASE study participants who consented and who got screened for lung cancer.

	Consented Participants		Screened Participants		
Demographics of enrolled participants	*N* = 257	%	*N* = 106	%	*p*‐value
Age, years					
Mean (SD)	62.7 (6.7)		63.7 (7.1)		0.44
Median (min–max)	62.0 (50.0–84.0)		63.0 (50.0–84.0)		
Age categories					
50–59	86	33.5	33	31.1	0.001
60–69	114	44.4	46	43.4	
70–79	41	16.0	26	24.5	
80–89	2	0.8	1	0.9	
Missing	14	5.4	‐	‐	
Gender					
Men	109	42.4	57	53.8	0.0027
Women	146	56.8	49	46.2	
Missing	2	0.8			
Race					
Black/African American	223	86.8	89	84.0	0.21
Caucasian	30	11.7	17	16.0	
Hispanic/Latino	1	0.4	‐	‐	
Native American	1	0.4	‐	‐	
Missing	2	0.8	‐	‐	
Health Insurance					
No	27	10.5	3	2.8	0.0006
Yes	222	86.4	101	95.3	
Missing	8	3.1	2	1.9	
Insurance Coverage					
Medicare	116	45.1	54	50.1	0.12
Medicaid	42	16.3	23	21.7	0.02
Private	57	22.2	24	22.7	0.88
Other	31	12.1	18	17.0	0.04
None	3	1.2	1	0.9	0.78
County					
Choctaw	29	11.3	22	20.8	
Dallas	32	2.5	5	4.7	
Greene	7	2.7	3	2.8	
Hale	5	2.0	1	0.9	
Jefferson	127	49.4	60	56.6	
Marengo	1	0.4	10	9.4	
Sumter	26	10.1	4	3.8	
Missing	4	1.6	1	0.9	
Urban/rural					
Urban	127	49.4	60	56.6	0.32
Rural	126	49.0	45	42.5	
Missing	4	1.6	1	0.9	

*Note*: *p*‐value compares proportion between those who got screened for lung cancer compared to those did not.

Of the total consented (*n* = 257), 86.4% of the participants had health insurance coverage (*n* = 222) with most of them under the Medicare coverage (*n* = 116).

A total of 106 completed lung cancer screening (106/257, 41%) (Table 1). The mean age of those screened was 63.7 (±7.2); 54% were males, and 84% were African American or Black. About 57% were from the urban county while 43% were from rural county. Almost 95% had insurance coverage with 50% with Medicare, 22% with Medicaid and 23% with Private insurance. Demographic characteristics of persons screened were similar as those who did not screen with respect to age and race/ethnicity; however, a larger proportion of men (54% vs. 35%, *p* = 0.001) and persons with health insurance (95% vs. 83%) completed screening when compared to those who did not complete screening. Screening results of study participants found that seven participants received a lung cancer diagnosis; 33 required a follow‐up within 3–6 months, and 63 required screening annually (no reported in a table).

Many of the study participants acknowledged their need for more education and awareness mostly on cancer and the screening procedures, while their healthcare and location needs were mostly centered on needing assistance with navigating through clinics and having healthcare facilities close to their residence (Table 2).

**TABLE 2:** Needs identified about lung cancer screening among participants enrolled in the study.

Needs	Consented Participants	
	*N* = 257	%
Education/Awareness		
Needs more info about the cancer	75	29.2
Needs to know more about screening procedures	43	16.7
Needs to know how to get help	23	8.9
Needs written info that is easily understood	3	1.2
Needs help understanding doctor's instructions	7	2.7
Needs guidance throughout screening and treatment	15	5.8
Needs to know about screening/treatment and follow‐up is kept confidential	4	1.6
Would like help with filling out forms and understanding written info about treatments and cancer	3	1.2
Needs to talk to someone who has been through the experience of cancer	3	1.2
Healthcare Experience		
Needs help to get through the maze and confusion of clinics	23	8.9
Hospital is too confusing	3	1.2
Needs doctor to take time to explain medical condition	8	3.1
Needs to be seen in a timely manner	5	1.9
Needs help understanding what doctor needs him/her to do	4	1.6
Facility location		
Needs clinic/screening facility/clinic close to residence	41	16.0
Needs reliable transport to and from appointments	28	10.9
Financial		
Needs insurance	12	4.7
Needs to be able to pay for treatment	7	2.7
Needs to know that screening/treatment/follow‐up will be paid for	8	3.1
Needs to know about insurance and coverage for cancer screening and treatment	4	1.6
Needs to know more about Medicare/Medicaid benefits	2	0.8
Social		
Worried about what family may say	7	2.7
Worried about how family/friends may feel	4	1.6
Needs someone to help watch children/other relatives	1	0.4
Needs help keeping up with appointments	47	18.3
Needs family more involved and supportive about cancer related concerns	7	2.7

About 121 participants reported one or more barriers for screening (Table 3). These included transportation, emotional concerns, COVID concerns, financial issues, health issues such as arthritis, or fear of findings. Very often, no reason was given when the coordinators called the participants to follow‐up about their primary care visit. There were no significant differences in barriers experienced by those who were screened compared to those who were not screened.

**TABLE 3:** List of barriers experienced by ALCASE study participants during the study (enrolled vs. screened).

Barriers/Issues	Consented participants *N* = 257		Participants with screening results *N* = 106		*p*‐value
	*N*	%	*N*	%	
Total people who reported any barrier	121	47.1	68	64.2	<0.001
Transportation	19	7.4	13	12.5	0.48
Family issues	7	2.7	3	2.9	0.14
Financial issue	12	4.7	7	6.7	0.69
Healthcare	10	3.9	4	3.8	0.30
Eligibility requirement	6	2.3	3	2.9	0.78
Job/work issues	2	0.8	2	1.9	0.20
Emotional concerns	16	6.2	8	7.7	0.63
Phone issues	1	0.4	1	1.0	0.37
COVID	12	4.7	5	4.8	0.31
Other^a^	72	28.0	38	36.5	0.99

*Note*: *p*‐value compares proportion between those who got screened for lung cancer compared to those did not.

^a^ Fear of findings, health issues like arthritis, or no reason was provided.

## 4 RESULTS INTERVIEWS

The sample consisted of five participants—three county coordinators, one case manager, and one program manager (Table 4). Four themes emerged from the interviews (Table 5).

**TABLE 4:** Demographic characteristics of ALCASE staff.

ALCASE staff	Gender	Race	Years of experience
Program Manager	Female	African American	30
Case Manager	Female	African American	20
County Coordinator 1	Female	African American	20
County Coordinator 2	Female	African American	15
County Coordinator 3	Male	African American	2

**TABLE 5:** Table of themes from the debriefing interviews.

Themes	Sub‐themes
Overall impressions of implementing ALCASE were positive	(a) The project was helpful in not only leveraging existing infrastructure but also in expanding early detection programs especially since lung cancer is the leading cause of cancer death in Alabama (b) The project required extensive planning and dedicated personnel
Barriers in implementing ALCASE were identified predominantly at the physician and institutional level	(a) Lack of PCP awareness was a hindrance in implementing the ALCASE project. (b) Lack of adequate screening centers in the state of Alabama impeded high‐risk individuals from getting screened (c) The pandemic compromised the execution of the project ALCASE
Facilitators in implementing ALCASE were identified at multiple levels	(a) Ease of LCS utilizing LDCT was an added advantage in persuading screen eligible individuals to undergo screening (b) The program would have benefitted from a centralized LCS program (c) Personalized incentives can help facilitate participation in projects such as ALCASE
Suggestions on improving LCS leaned toward mitigating barriers at the institutional and structural level	(a) Strategic involvement of other health care systems would aid in increasing screening (b) Future projects should increase scope of work to account for extensive navigation (c) Continued community outreach about disclosing smoking status, long‐term effects of smoking and dissemination of current project would foster further engagement with early detection programs

### 4.1 Theme 1: Overall impressions of implementing ALCASE were positive

ALCASE project staff stated that it was a project worthy of pursuing, given that lung cancer was a leading cause of cancer death and there was added significance of implementing this project in rural counties of Alabama. The program manager stated that the project presented an opportunity to not only leverage the existing infrastructure but to also expand on it.

ALCASE itself was a good project because it allowed our infrastructure to expand to lung cancer screening. So, we spent a lot of time in developing and getting the content together to do the CHA child training. So, the processes had multiple layers, one training of the CHA then utilizing the CHA and the county coordinators to educate and identify people like they did for the other cancers in their communities. I think it was a great project for us to learn all of the modalities related to lung cancer. It also gave us a fresher perspective about how the community will respond to lung cancer screening, so that future programs could be adapted to what makes sense to the members of the community. (Program director)

ALCASE project staff discussed the necessity for getting involved in lung cancer screening especially due to the enormous mortality burden of lung cancer.

But we knew it was needed because they were saying, look it's the number one killer, you know, so it was needed (County‐coordinator‐1)

The case manager of the study stated that the project was carefully considered since there were personnel attached for every aspect of the lung cancer screening process.

I was hired and we started developing paperwork so that we could give it to the coordinators whenever they reach out to potential participants who want it to be screened. And it just started flowing from there, so we had so many documents to create. to be able to capture the information that you know we want it to capture and need it just in case that person was going to be screened. So, lots of paperwork, lots of forms to be created, and things of that nature. So that's where I actually got into making sure that we had everything we need when we start it to get out there in the field. (Case manager)

Another county coordinator discussed the lack of awareness regarding dangers of sustained long‐term smoking and reiterates the need for and the importance and impact of the program.

They don't understand how much smoking impacts them. As far as thinking about on a knowledgeable level like, guess they associate smoking equals cancer. They get that, but they don't understand the longevity of smoking. How long they've been smoking over the vast amount of years, how they can accrue up to them really needed to be urgent about seeing about their own lung care the lung health. (County coordinator‐4)

In summary, the ALCASE staff was appreciative of the lung cancer screening program in Alabama since it was not previously included in the outreach and engagement programs.

### 4.2 Theme 2: Barriers in implementing ALCASE were identified predominantly at the physician and institutional level

All ALCASE staff identified barriers at the provider and institutional level. Clinician endorsement, lack of lung cancer screening awareness among the clinicians in addition to logistical factors of establishing screening centers without adequate resources emerged as sub‐themes. Building relationships with clinicians was tough and made screening for lung cancer daunting. Although the staff was aware of a clinician component in lung cancer screening, relationship building with physicians in the primary care setting was hard to build and establish.

Yeah. So, we were aware that that component existed. But in doing so, our case manager, was responsible for building those relationships with the clinicians. And so how those, how that relationship was built required a lot of work and a lot of one‐on‐one interaction there, and each health care system was different. We didn't know the providers. The providers didn't know us and so somebody needed to be dedicated to work with the provider, so that we can have a pathway if you will, to send patients there. (Program Director)

In addition to establishing relationships, ALCASE staff also stated that undertaking this initiative took time and consequently depleted the time allotted to complete outreach and engagement activities.

I drove around to every doctor's office and presented them with this folder and talk with the nurses and the scheduling department to be able to get them on one accord with what we were trying to do out here in the field that probably took a probably close to a month to provide that you know that service, but it was well worth it, because we were being, you know, and I was being bombarded with calls like, Well, why are they coming in here? What are you all trying to do around here? So, we just introduced ourselves, so to speak, to the doctor. (Case Manager)

Establishing Screening Centers of Excellence within driving distance was difficult and required onerous amounts of paperwork that needed to be completed and was identified as an impediment when implementing the project.

But even among cancer screening centers, when you live in rural Greene County, the county and you telling me that I have to go to Birmingham, and I don't have transportation, or you tell me I have to go to Carmichael in Montgomery when you have regional hospitals.(County Coordinator‐1)

Another participant discussed that despite the institution's long‐standing presence in the community especially with regard to cancer preventive services, the staff associated with the project were faced with barriers when it was time to expand to lung cancer screening.

So even with all of our work that we've done so, we've been working in breast and cervical for over 25 years, and there are a lot of baby steps to where we are today that even today, we're having to revisit those relationships with the clinics, the rules, the guideline more importantly, the personnel. And so there are some people you will encounter that. No matter who you are, they're not gonna they're not gonna play in the sandbox with you, and you just have to know that now the tragedy is to the communities that they serve. (Program Director)

Lack of awareness regarding lung cancer screening guidelines was reported to be prevalent. One ALCASE staff member discussed that the primary care physicians were not aware of screening eligibility or the nuances regarding modality of lung cancer screening and had to be overcome with further education to the community of primary care physicians.

When you started to talk to people about lung cancer screening—they didn't know about the screening guidelines. They didn't know that it was such thing where they knew heard about. You people have lung cancer, but they didn't know they wasn't really educated on when to get the cancer screens. And we found that a lot of doctors were not knowledgeable. and so, we had to educate them. (County Coordinaor‐2)

The doctors didn't, you know they were like—Well, we're gonna do a chest X right first, and she said. My chest look fine, you know. So then we had to kind of go back to the drawing board, and we had to create a letter. We called it the dear doctor letter to explain that you know the proper way to screen for lung cancer was the low dose CT. Scan, so we provided them with a folder. (Case Manger)

On the contrary, COVID‐19 (while not an institutional or physician level barrier) was reported to be a major hindrance in implementing ALCASE. Given that a significant portion of the project was outreach and required Community Health Advisors (CHA) and County Coordinators to be out in the community, COVID restrictions played an enormous toll on the staff's ability to be involved in community engagement. It necessitated a change in how the project was implemented. All the in‐person education and awareness events needed to be conducted remotely.

We could have spent more time on the street talking to more people. But, with ALCASE in the middle of a pandemic—because everything was shut down. (Case Manager)

We did some education through Zoom. So, we made adjustments. And I think a lot of the information would have been better, had it been more face‐to‐face. (County coordinator‐1)

In summary, when staff were asked about barriers in implementing the ALCASE project, all participants (4) discussed barriers at the physician and the institutional level. The general perception was that patient activation was not a roadblock especially since CHA and County Coordinators had longitudinal relationship with the community and that mitigating institutional and physician level factors would be crucial and can help in increasing lung cancer screening rates in the Black Belt region of Alabama.

### 4.3 Theme 3: Facilitators in implementing ALCASE were identified at multiple levels

ALCASE staff identified many individual level factors that facilitated the program. ALCASE case manager stated the ease of getting screened for lung cancer was an added advantage and aided in increasing lung cancer screening.

We provided them with information on that brochure that says the screening process is so easy. No needles it only take about 5 min. You can leave your clothes on. You just walk in, and they you are usually in and out within about 15 to 20 min. (Case manager)

The same staff member discussed that a dedicated CHA could facilitate a program that aims to increase lung cancer screening knowledge by gamifying the education and thereby facilitate better education and retention of the information.

You know, ease into it what you want them to do, and present that information to them. So, we even have, like I might take the information and I might make a little game out of it. I might get a…you know, like a cart, board or something, and I might have a jeopardy board, or something like that. It takes some time, but they enjoy it, and they retain the information. (Case manager)

Another ALCASE staff discussed that when there is a comprehensive lung cancer screening program in a health care system, it aided in completion of lung cancer screening.

One time—they started with the PCP. And got their screen all in one. It was the one time shop, which was perfect, which was good because they just came one time. (County coordinator‐2)

In summary, facilitators identified by the ALCASE staff were seen at multiple levels. In addition to incentives, patient activation toward screening was related to having effective CHAs who can help not only in providing information but also in retaining it.

### 4.4 Theme 4: Suggestions on improving lung cancer screening leaned toward mitigating barriers at the institutional and structural level

ALCASE staff reflected on barriers to implementing ALCASE and had various suggestions on improving outreach and engagement toward lung cancer screening. Staff discussed being strategic about involving other health care systems who are dedicated partners in lung cancer screening programs.

My recommendation for a future project would only be to work with the facility which you have a MOU with, and go in upfront with what the expectations are and that kind of thing. (Program Director)

One county coordinator discussed the navigation component that was not previously accounted for in the coordinator's scope of work and stated that it needs to be included for all future lung cancer screening projects.

The initial structure, from what I've understood of our case was that for each county there was one coordinator. but there was a lot of work that ended up on the Coordinator because of all the navigation and all of the back and forth, and all of the keeping tabs on every patient or every address. (County coordinator‐2)

The ALCASE case manager suggested finding an opportunity to bring the results back community stakeholders (e.g., participants and medical providers) in order to close the loop and discuss future plans of continuing to work in the lung cancer screening space,

At the end of that you need to bring all of those people back. Have them a little snack or a dinner or something. Thank them for participating, and give them the stats. What did you find from this? (Case manager)

One county coordinator stated that community members were not being honest about pack years of smoking with the PCPs. This was a lesson learnt while educating high‐risk individuals regarding lung cancer screening and which needs to be incorporated into the curriculum.

So educating them to be honest about their pack years. Be honest about, you know any symptoms, so they could tell the doctor. When she came back, she was like her doctor said that she couldn't get it. I'm like Well, did you tell them that your pack years? And she was like I was embarrassed if I haven't had any cigarette picks that I smoke per day. And so what? That's why they denied you from getting your screening, you know, going to the next step, because you have to tell them true. Tell them a beat about your pack years like, Be honest with them because we were thinking they're gonna go in the office and they go and tell the doctors everything. (County coordinator‐2)

A county coordinator discussed the importance of including those who don't have insurance to get screened for lung cancer by getting the PCPs involved in accepting project sponsored vouchers.

If I'm saying is if I say I didn't have insurance, then maybe I should've a voucher and the docs had agreed to accept. The law could give you this brochure and you would go to the doctor's office and then they pay for that. Rather than me, I had to go in my pocket and give you towards a screening. (County coordinator‐1)

Finally, another staffer discussed personalized incentives that support high‐risk individuals getting screened for lung cancer considering distance to screening centers varied depending on the individual's county of residence.

Transportation needs and gas cards need to be more personalized. It cannot be a flat fee kind of the thing. That's where they come from‐ Anderson, Meridian, Mississippi! (County coordinator‐2)

In summary, when ALCASE staff were asked about suggestions to improve the program, they stated that mitigating some of the institutional factors that impede lung cancer screening would help in increasing screening rates. Additionally, the staff suggested that utilizing community engaged participatory principles of ensuring results of the current project would help in continuing lung cancer screening in the community.

## 5 DISCUSSION

The goal of ALCASE was to provide education, screening awareness and for CHAs to identify 250 screen eligible individuals across seven counties and navigate them to be screened. Our coordinators assumed the role of lay navigators and navigated 106 high‐risk individuals to lung cancer screening using LDCT. Navigation activities included talking to individuals in the community setting, providing one‐on‐one education, assessing patient barriers to screening such as transportation, enrolling patients into health insurance programs; facilitating access to primary care providers, helping patients resolve barriers—such as patient reminders, follow‐up, and tracking. This is consistent with other studies describing the time‐intensive delivered activities as reported by navigators.^22^, ^23^, ^24^ Due to the pandemic restrictions, our staff had to be resourceful in outreach and engagement approaches. Our in‐person education was transferred to text messages, direct mailing, conference calls, and video conferences.^25^ We were able to reach 447 participants and 257 consented and expressed interest in lung cancer screening. This is noteworthy since previous literature discusses motivating smokers to participate in lung cancer screening is complex and when coupled with stigma, the target population for lung cancer screening of current and former smokers is widely considered as hard to reach and requires consistent investment in strategies that can increase lung cancer screening awareness.^26^

One of the biggest barriers as reported by the ALCASE staff to getting screened for lung cancer was the lack of screening centers within drivable distance (less than 60–90 minutes each way) which was reiterated as lack of transportation by our participants as well. This in part is also the reason for our slightly higher percentage of individuals from our urban county who have access to a SCOE within the county. This is concerning considering that screening centers are geographically maldistributed^27^ relative to the rural–urban and regional need and calls for models of care that are more disposed toward rural dwellers.^26^, ^27^

Our staff reported another critical barrier to lung cancer screening rates—the lack of awareness among primary care physicians. Because of the physician's critical role in engaging high‐risk individuals in screening and ordering lung cancer screening—this finding is important to note. Additionally, it adds to the growing body of literature^28^, ^29^, ^30^, ^31^ that demonstrates physician knowledge continues to be sub‐optimal especially with the recent changes to USPSTF recommendations.

Our coordinators discussed a facilitator of note—the presence of a centralized lung cancer screening program. The centralized model uses a dedicated program coordinator who verifies the eligibility of referred patients, conducts shared decision making, provides tobacco cessation support, and oversees the process from ordering the LDCT scan to scheduling appropriate follow‐ups, including annual examinations.^32^ In contrast, patients in a decentralized model are managed primarily by their clinicians.^32^ This finding is of consequence to future efforts of increasing lung cancer screening rates since recent studies have shown that decentralized programs are inferior to centralized programs both in terms of screening rates and screening adherence after the first screening.^33^, ^34^

Our study highlights the importance of promoting awareness regarding long‐term smoking effects. Our coordinators also stated that individuals were not honest about their smoking status. This is consistent with previous research that shows smoking‐related stigma is associated with deleterious health behaviors, including a reluctance to seek cessation assistance and a tendency to conceal smoking behaviors.^35^, ^36^ Furthermore, this finding has larger implications on how CHAs conduct these conversations with community members. A recent study has demonstrated that these CHAs—widely regarded as “change agents,” may greatly benefit from smoking cessation training sessions that are better tailored to the way they provide health education to communities, and that the tailored training curriculum should highlight the specific needs of more vulnerable populations who are most likely to engage with CHAs.^37^, ^38^

This project is not without limitations. We were unable to incorporate smoking‐related stigma and lung cancer stigma into our education curriculum.^14^ Stigma indubitably adds complexity to lung cancer screening participation. This will need to be addressed in future studies especially since stigma is highly prevalent, affects healthcare engagement, and consequently affects public attitudes, policy decisions, media campaigns, and research funding.^26^, ^39^ Additionally, we did not offer smoking cessation since it was beyond the scope of the project. While there is a strong rationale for providing smoking cessation interventions for individuals who smoke and are undergoing lung cancer screening, lack of effective smoking cessation strategies currently hinder realizing the full benefits of lung cancer screening.^40^ Additionally, we did not incorporate the patient perspective of the program. Similarly, this evaluation didn't include the providers' perspective. Also, we were unable to include all ALCASE staff members due to staff attrition. Balancing these limitations are several strengths. Firstly, our project aligned with the priority of our cancer center to reach harder to reach populations. We leveraged our long‐standing Deep South Network (of over 20 years) that facilitated identification of CHAs, provided access to community—allowing access to our priority populations of racial/ethnic minority and medically underserved individuals. As a result of this longitudinal relationship, we were able to navigate about 41% of individuals from being identified as interested in screening to getting screened—far surpassing the national average rate of lung cancer screening.^9^, ^10^ Additionally, this enduring relationship can in part explain the lack of differences in our data by those who reside in rural versus urban areas. Secondly, unlike previously reported lack of recruitment strategies and bias toward recruiting men, people from higher socio‐economic backgrounds to lung cancer screening, we were able to engage with and recruit rural minoritized, underserved individuals who are current and former smokers.^41^ This highlights the significant role that CHAs/County coordinators can play in the US screening programs, especially since program managers report having limited information about people at high risk within the local population and how best to recruit potential participants.^42^

## 6 CONCLUSION

This program leveraged an existing infrastructure to implement a lung cancer screening program to reach socioeconomically disadvantaged community and minoritized individuals. While patient activation was possible, the program also allowed us to explore some of the barriers such as limited healthcare provider and health system engagement. Our future endeavors will continue to focus on empowering Alabamians with information around cancer preventive screenings, increasing cancer referrals and screening rates and in doing so make a notable impact on the devastating toll that lung cancer continues to have in Alabama.

## AUTHOR CONTRIBUTIONS

**Soumya J. Niranjan:** Investigation (equal); writing – original draft (equal); writing – review and editing (equal). **Meghan Tipre:** Data curation (equal); formal analysis (equal); investigation (equal); project administration (equal); writing – review and editing (equal). **Claudia M. Hardy:** Project administration (equal); writing – review and editing (supporting). **Tara Bowman:** Data curation (equal); project administration (equal). **Monica L. Baskin:** Conceptualization (equal); funding acquisition (equal); methodology (equal); writing – original draft (equal); writing – review and editing (equal).

## CONFLICT OF INTEREST STATEMENT

No conflicts of interest to report.

## Supporting information


Data S1.

## Data Availability

The data that support the findings of this study are available on request from the corresponding author. The data are not publicly available due to privacy or ethical restrictions.
