# Supplementary material for: Empowering minoritized Alabamians screened for lung cancer—The Alabama Lung Cancer Awareness Screening and Education (ALCASE) project
Source: Cancer Med. 2024 Oct 14;13(19):e70213. doi: 10.1002/cam4.70213 (PMC11472649; doi:10.1002/cam4.70213)
Supplement: Supplementary file 1 — Data S1. [file CAM4-13-e70213-s001.docx]

De-brief questions for ALCASE study coordinators

1. What was your general experience when talking to people about lung cancer screening?

- Probe: Were they apprehensive / uneasy while talking about their smoking status and / or screening for lung cancer?

1. What was your overall impression of the program - ALCASE ?
2. What aspect of the program worked best?

- Probe: What did you like about working in this program?

1. What aspect of the program needs improvement?

- Probe: What can we do better?

1. If you could change anything about the program, what would you change?
2. How can we reach more people and how can we convince more people to get screened?
